# Quantification of Iris Atrophy by Swept-Source Optical Coherence Tomography in Posner–Schlossman Syndrome

**DOI:** 10.3390/jcm11216484

**Published:** 2022-11-01

**Authors:** Xiaoqin Yan, Mu Li, Wei Chen, Zhiqi Chen

**Affiliations:** 1 Department of Ophthalmology, Tongji Hospital, Tongji Medical College, Huazhong University of Science and Technology, Wuhan 430000, China; 2 Department of Ophthalmology, Union Hospital, Tongji Medical College, Huazhong University of Science and Technology, Wuhan 430000, China

**Keywords:** Posner-Schlossman syndrome, iris atrophy, optical coherence tomography

## Abstract

**Purpose**: To investigate iris atrophy in Posner–Schlossman syndrome (PSS). **Methods**: Sixty-one patients with PSS were included. Using swept-source optical coherence tomography, the thickness of anterior border layer (A), middle stromal layer (M), and the posterior pigmented epithelial layer (P) of iris were measured at 500 μm, 1000 μm, 1500 μm, 2000 μm, and 2500 μm from the pupillary edge in both PSS-affected and fellow eyes. The relationships between iris thickness and corneal endothelium density, cup-to-disc (C/D) ratio, and retinal nerve fiber layer (RNFL) thickness were also estimated in PSS-affected eyes. **Results**: Iris thickness parameters, including M500, M1000, A1500, A2000, P2000, and P2500, were significantly thinner in PSS-affected eyes than in fellow eyes (all *p* < 0.05). Moreover, M500 and M1000 were significantly associated with corneal endothelium density (*p* = 0.047 and 0.018, respectively), and M500 was significantly associated with C/D ratio (*p* = 0.001) and RNFL thickness (*p* = 0.037) in PSS-affected eyes. **Conclusions**: Iris showed significant thinning and atrophy in PSS-affected eyes, and iris stromal thickness close to the pupillary edge could be a novel clinical predictor of the changes in corneal endothelium, C/D ratio, and RNFL thickness in PSS.

## 1. Introduction

Posner–Schlossman syndrome (PSS) is a special type of anterior uveitis that was first reported in 1948 [1]. An epidemiological study of China showed that the annual incidence of PSS could approach to 3.91 per 100,000 persons, and the cumulative incidence of PSS could be 39.53 per 100,000 persons over 10 years [2]. Although the exact etiology of PSS was still elusive, recent studies have suggested that inflammatory cytokines [3,4,5,6], cytomegalovirus (CMV) infection [7,8,9], vascular endothelial dysfunction [10], and gene susceptibility [11] might be associated with the pathogenesis of PSS.

PSS is clinically characterized by recurrent and acute attacks of unilateral, relatively mild, non-granulomatous anterior uveitis, and elevated intraocular pressure (IOP) [1,12]. In addition to that, other clinical features, e.g., iris atrophy, keratic precipitates (KPs), and rare posterior synechiae, were also reported in PSS [13,14]. Previously, PSS has supposed to have been benign because only the vision of PSS patients was mildly affected, while the visual field and optic disc of PSS patients still remained normal [1]. However, corneal endothelial cell loss and glaucomatous optic nerve injury were reported in PSS in recent studies [7,15,16], suggesting that the impact of intraocular hypertension and secondary glaucoma in PSS has probably been underestimated [17].

Among the various features of PSS, Kim et al. reported that iris involvement (iris atrophy, iris depigmentation, iris thinning) in PSS could be a significant clinical predictor of the requirement for anti-glaucoma surgical intervention, as well as of the worse vision-related quality of life questionnaire scores [18]. Although several studies have investigated iris involvement in PSS by slit lamp microscope qualitatively [13,14,18,19,20,21,22,23], no studies have performed quantitative measurements of iris dimensions in PSS for now. Considering the important role of iris involvement in PSS [18], quantitative measurements of iris dimensions might provide us with novel information about PSS.

With the development of optical coherence tomography (OCT), it could provide detailed and clear observation and accurate quantitative measurements of the ocular anterior segment biometrics, including the iris [24,25,26]. Accordingly, using swept-source OCT (SS-OCT), this study aimed to compare iris thickness between the affected and fellow eyes in patients with PSS and to investigate the associations between iris thickness and other PSS-affected parameters, e.g., corneal endothelium density, cup-to-disc (C/D) ratio, and retinal nerve fiber layer (RNFL) thickness.

## 2. Materials and Methods

This observational study was approved by the ethics committee of Tongji Hospital, Huazhong University of Science and Technology (TJ-IRB20201024) and was in adherence to the tenets of the Declaration of Helsinki. Written informed consent of study subjects were signed before participation.

### 2.1. Subjects

Sixty-one patients with PSS were included in this study. All of them received ophthalmic examinations, including slit lamp examination, gonioscopy, corneal endothelial microscope, fundus examination, RNFL thickness measurement by SS-OCT (VG200S; SVision Imaging, Henan, China), C/D ratio assessment with fundus photographs (AFC-210, Nidek Co., Ltd., Gamagori, Aichi, Japan) by two glaucoma specialists (WC and ZC), axial length (AL) measurement (IOL-Master 500, Carl Zeiss Meditec, Dublin, OH, USA), and IOP measurement (Goldmann tonometry, Keeler Ltd., Windsor, UK). The diagnostic criteria of PSS were: (1) unilateral and recurrent disease attack; (2) transient IOP elevation with blurred vision; (3) hoar and white suet-shaped KPs and/or mild inflammation in the anterior chamber; (4) open anterior chamber angle without iris synechiae [27]. Patients with a history of ocular surgery or systemic diseases were excluded from this study.

### 2.2. SS-OCT Imaging Acquisition and Processing

Both the affected and fellow eyes of the recruited patients with PSS underwent SS-OCT examinations (CASIA SS-1000, Tomey Corporation, Nagoya, Japan). All the eyes were imaged by the same experienced examiner (XY). Both the nasal and temporal limbi were scanned separately after adjusting the fixture to the corresponding zones.

**Figure 1:**
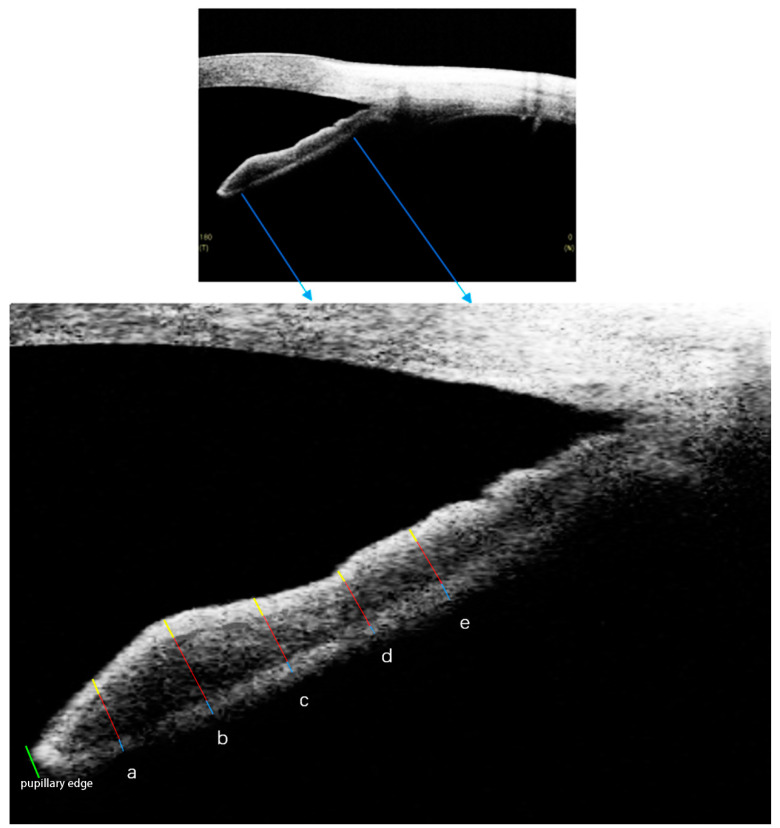
The measurements of iris thickness. The anterior border layer (yellow line) and posterior pigmented epithelial layer (blue line) of iris are seen as two separate narrow hyper-reflective bands. The middle stromal layer (red line) of iris is seen as a wide hypo-reflective band. The iris thickness was measured at 500 μm (a), 1000 μm (b), 1500 μm (c), 2000 μm (d), and 2500 μm (e) from the pupillary edge (green line), respectively.

The iris was divided into three sublayers: the anterior border layer, the middle stromal layer, and the posterior pigmented epithelial layer. Among them, the anterior border layer and the posterior pigmented epithelial layer were narrow hyper-reflective bands, while the middle stromal layer was wide hypo-reflective band in the OCT images [26]. Optimal image magnification and contrast were subjectively defined to maximize the three sublayers of iris. The thickness of each sublayer of iris was measured at 500 μm, 1000 μm, 1500 μm, 2000 μm, and 2500 μm from the pupillary edge (Figure 1). Thus, we collected iris thickness data as follows: the anterior border layer thickness at 500 μm (A500), 1000 μm (A1000), 1500 μm (A1500), 2000 μm (A2000), and 2500 μm (A2500) from the pupillary edge; the middle stromal layer thickness at 500 μm (M500), 1000 μm (M1000), 1500 μm (M1500), 2000 μm (M2000), and 2500 μm (M2500) from the pupillary edge; and the posterior pigmented epithelial layer thickness at 500 μm (P500), 1000 μm (P1000), 1500 μm (P1500), 2000 μm (P2000), and 2500 μm (P2500) from the pupillary edge. The pupil diameter (PD) was measured as the distance from one side to the opposite side of the pupillary tip of the iris [28].

All the measurements were performed using ImageJ software (National Institutes of Health, Bethesda, MD, USA) by the experienced observer (ML), and all the measurements were masked to the study subject information. Iris thickness and PD were re-measured by the same experienced observer (ML) in a separate session to test the measurement reproducibility.

### 2.3. Statistical Analysis

Data were presented as mean ± standard deviation where applicable. Differences between PSS-affected and fellow eyes were compared using paired *t*-test. Parameters with nasal and temporal measurements were compared using generalized estimating equations (GEEs) because GEEs took the correlation between the measurements from the nasal and temporal quadrants of one eye into account. Linear regression was used to determine the associations between iris thickness and corneal endothelium density, C/D ratio, RNFL thickness, and IOP. Adjusted *β* coefficients for the associations between the independent and dependent variables were assessed using GEEs. The intra-observer reproducibility was assessed with the intraclass correlation coefficient (ICC). All analyses were conducted using the R software version 3.4.3 (http://www.r-project.org (accessed on 5 March 2022)). All tests were two-tailed, and statistical significance was defined as *p* value of <0.05.

## 3. Results

The mean age of the study subjects was 36.56 ± 11.80 years. Among them, 34 of 61 (55.74%) were male, and 27 of 61 (44.26%) were female. There were no significant AL differences between PSS-affected and fellow eyes (23.88 ± 0.95 vs. 23.87 ± 0.91 mm, *p* = 0.848). Meanwhile, the corneal endothelium density (2447.70 ± 423.93 vs. 2706.43 ± 297.06/mm^2^, *p* < 0.001), C/D ratio (0.50 ± 0.19 vs. 0.37 ± 0.09, *p* < 0.001), RNFL thickness (94.56 ± 23.34 vs. 101.72 ± 10.38 μm, *p* = 0.002), IOP (23.71 ± 11.96 vs. 17.43 ± 3.74 mmHg, *p* < 0.001), and PD (4.63 ± 0.88 vs. 4.43 ± 0.89 mm, *p* = 0.032) showed significant differences between PSS-affected and fellow eyes (Table 1).

Comparisons of iris thickness between PSS-affected and fellow eyes.

**Figure 2:**
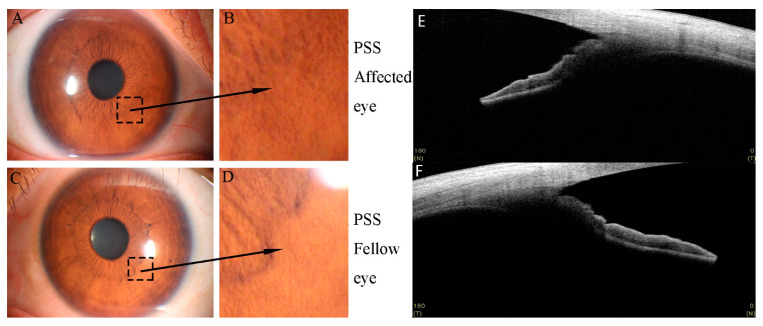
The iris of PSS-affected and fellow eyes (observed by slit lamp microscope: (**A**–**D**) and by SS-OCT: (**E**,**F**). Compared with PSS fellow eye (**C**,**D**,**F**), PSS-affected eye (**A**,**B**,**E**) showed significant iris atrophy.

**Figure 3:**
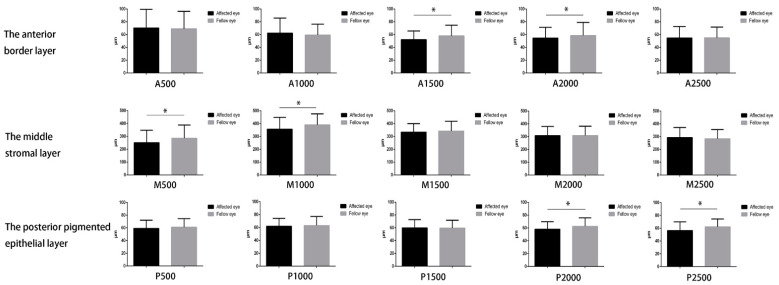
Comparisons of iris thickness between PSS-affected and fellow eyes. * Significance of difference by generalized estimating equations (the influence factors as age, sex, AL, PD, and IOP have been adjusted). A: The anterior border layer, M: the middle stromal layer, P: the posterior pigmented epithelial layer.

M500, M1000, A1500, A2000, P2000, and P2500 of PSS-affected eyes were significantly thinner than those of fellow eyes (all *p* < 0.05) (Figure 2 and Figure 3).

### 3.1. Univariate Linear Regression of Associations of Iris Thickness and Corneal Endothelium Density in PSS-Affected Eyes

In PSS-affected eyes, M500 and M1000 were significantly and positively associated with corneal endothelium density (*p* = 0.047 and 0.018, respectively), while the rest of iris thickness parameters showed no significant associations with corneal endothelium density (all *p* > 0.05) (Table 2).

### 3.2. Univariate Linear Regression of Associations of Iris Thickness and C/D Ratio in PSS-Affected Eyes

M500 was significantly and negatively associated with C/D ratio (*p* = 0.001), while no other iris thickness parameters showed significant associations with C/D ratio (all *p* > 0.05) in PSS-affected eyes (Table 3).

### 3.3. Univariate Linear Regression of Associations of Iris Thickness and RNFL Thickness in PSS-Affected Eyes

M500 was significantly and positively associated with RNFL thickness (*p* = 0.037), while no other iris thickness parameters showed significant associations with RNFL thickness (all *p* > 0.05) in PSS-affected eyes (Table 4).

### 3.4. Univariate Linear Regression of Associations of Iris Thickness and IOP in PSS-Affected and Fellow Eyes

No significant associations between iris thickness and IOP were found in both PSS-affected and fellow eyes (Table 5).

The reproducibility of iris thickness and PD measurements.

**Figure 4:**
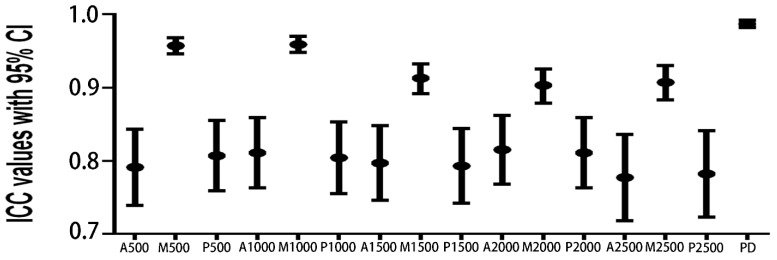
The intra-observer reproducibility of iris thickness and pupil diameter measurements. ICC: Intraclass correlation coefficient. CI: confidence interval. A: The anterior border layer, M: the middle stromal layer, P: the posterior pigmented epithelial layer. PD: Pupil diameter.

Iris thickness and PD were re-measured by the same experienced observer (ML) in a separate session, and the results showed that the reproducibility of our measurements in this study was good. The ICC values ranged from 0.777 to 0.987 (Figure 4).

## 4. Discussion

Morphological observations of iris in PSS by slit lamp microscopy have been reported previously [13,14,18,19,20,21,22,23]. However, no study has measured the iris dimensions in PSS quantitatively for now. In this study, we performed quantitative measurements of iris thickness in different locations (500/1000/1500/2000/2500 μm from the pupillary edge) using SS-OCT. Moreover, we divided the iris into three sublayers, the anterior border layer, the middle stromal layer, and the posterior pigmented epithelial layer, to study the changes of each layer in PSS. The results showed that M500, M1000, A1500, A2000, P2000, and P2500 of PSS-affected eyes were significantly thinner than those of fellow eyes. Among them, M500 and M1000 were significantly associated with corneal endothelium density, and M500 was significantly associated with C/D ratio and RNFL thickness in PSS-affected eyes.

Previous studies have suggested that iris atrophy could be observed by slit lamp microscope in PSS [13,14,18,19,20,21,22,23]. Accompanied with iris atrophy, significant pupil dilation could also be observed [18,29], which is consistent with our study results (PSS-affected eyes had thinner iris thickness and larger PD). In this study, we observed significant atrophy of the middle stromal layer of iris at 500 μm and 1000 μm from the pupillary edge, which were also the locations of pupillary sphincter. Thus, we speculated that the thinning of M500 and M1000 might also indicate the atrophy of pupillary sphincter, which could consequently result in the observed larger PD in the PSS-affected eye [26]. In terms of the importance of iris thinning and atrophy in PSS, Kim et al. suggested that iris involvement could be a significant clinical predictor of the requirement for anti-glaucoma surgical intervention, as well as of the worse vision-related quality of life questionnaire scores. In addition, iris thinning and atrophy might also be a clinical clue for the increased number of PSS attacks, which could further damage the corneal endothelium and optic nerve [18].

Besides iris atrophy, PSS-affected eyes also showed lower corneal endothelium density in this study. Moreover, iris stromal thickness close to the pupillary edge (M500 and M1000) was found to be significantly and positively associated with corneal endothelium density in PSS-affected eyes, indicating that iris stromal thickness close to the pupillary edge might be a clinical predictor of corneal endothelium density loss in PSS. A previous study has reported that in PSS-affected eyes, the higher prevalence of iris changes was always accompanied by the more serious corneal endothelium loss [20], which was consistent with our study results. Iris stroma was susceptible to CMV [21] and could be a reservoir for the latency of CMV [30,31]. CMV has been identified to be the leading cause of PSS [7,8,9], and iris stromal atrophy is a distinctive characteristic of CMV anterior uveitis (e.g., CMV-positive PSS) [18]. The anterior uveitis caused by CMV infection could lead to the atrophy and thinning of iris as a result of direct viral invasion or vasculitis, as Woo et al. suggested [23]. In addition to anterior uveitis, CMV infection could also cause corneal endothelitis, leading to the decrease in corneal endothelium density and thickness [32,33]. This was also confirmed by the study of Su et al., reporting that patients with CMV-positive PSS suffered more corneal endothelium loss, compared with those with CMV-negative PSS [7]. In addition to CMV infection, inflammation might also play a role in the iris atrophy and corneal endothelium loss in PSS. PSS had significant elevated inflammatory cytokines and proteins in the anterior chamber [3,4,5,6]. In terms of iris, it is rich in blood vessels, and thus susceptible to inflammation. Aketa et al. reported significant associations between iris atrophy and inflammatory cytokines in the anterior chamber, indicating that iris atrophy was related to the increased levels of cytokines IL-1, IL-4, IL-6, IL-8, MCP-1, and TNF-α [34]. In terms of corneal endothelium, the anterior uveitis and anterior segment inflammation could also result in significant corneal endothelium loss [35]. Thus, the high level of inflammatory cytokines in the anterior chamber might also contribute to the iris atrophy and corneal endothelium loss in PSS-affected eyes.

In addition to the changes in corneal endothelium, larger C/D ratio and thinner RNFL were also observed in PSS-affected eyes in this study. Moreover, iris stromal thickness close to the pupillary edge (M500) was suggested to be significantly and negatively associated with C/D ratio and significantly and positively associated with RNFL thickness in PSS-affected eyes, indicating that the iris stromal thickness close to the pupillary edge might also be a clinical predictor of the optic cup enlargement and RNFL thinning (glaucomatous optic nerve injury). In primary angle closure glaucoma (PACG), the retinal vessel density in the affected eyes was significantly lower than that in the fellow eyes during the acute period [36,37]. Similar to the acute attack of PACG, PSS could also have acute elevation of IOP (40 mmHg or higher) [1,12], making the retinal vessel density of PSS-affected eyes significantly lower than that of PSS fellow eyes [38]. In addition to elevated IOP, the reduced optic nerve head and retinal perfusion is also closely associated with the progression of glaucoma [39,40,41,42]. The optic nerve head and retinal perfusion were determined by both the local arterial blood pressure and IOP [43]. The acute elevation of IOP in PSS could shrink the fundus blood vessels and reduce the fundus blood perfusion, leading to the glaucomatous optic nerve injury [38]. Similar to the retina, iris was rich in blood vessels, making it susceptible to the changes in IOP. When IOP increased acutely during PSS attacks, the blood perfusion of iris might decrease, resulting in the ischemia and atrophy of iris. Accordingly, the repeated attacks of PSS and elevation of IOP could be one reason for the changes in iris, C/D ratio, and RNFL thickness in PSS-affected eyes.

Aqueous humor obstruction is the major reason for the elevation of IOP. The main outflow pathway of aqueous humor is the conventional trabecular meshwork–Schlemm’s canal pathway [44]. Yan et al. have reported that the trabecular meshwork edema might play a role in the elevation of IOP in PSS [27]. However, besides the trabecular meshwork–Schlemm’s canal pathway, iris crypt could also drain aqueous humor. Iris is permeable and sponge-like, with the ability to alter its area and volume under various conditions [45]. Free access of aqueous humor into the loose connective tissue of the iris stroma has been observed, indicating that the iris pathway is also involved in the outflow of aqueous humor and the regulation of IOP [26,46,47]. A previous study by Chen et al. has indicated that the thinning of iris may result in the interferences in aqueous humor outflow through the iris, and further contribute to the elevation of IOP [26]. In this study, we observed significant iris thinning and atrophy in PSS. Accordingly, we speculated that in addition to the trabecular meshwork edema, iris thinning and atrophy might also contribute to the elevation of IOP in PSS.

Fuchs’ uveitis mimics PSS and can have high IOP, while the major difference is rubella titer [48]. Similar to PSS, iris atrophy is also reported in Fuchs’ uveitis; Fuchs’ uveitis had similar clinical findings as PSS, including mild unilateral anterior chamber inflammation with stellate KPs. It follows the course of a chronic, low-grade anterior uveitis. Iris atrophy and heterochromia are sometimes present, and early cataract is often observed in Fuchs’ uveitis. Due to these similar clinical features, PSS may be clinically misdiagnosed as Fuchs’ uveitis [4].

This study has certain limitations. First, most of our study subjects were under IOP-lowering medication treatment, which had a potential influence on the ocular blood flow, including the blood flow of iris [49]. Second, this was a cross-sectional study, not longitudinal. We did not follow-up with the patients, and thus did not observe the dynamic changes in iris during the full disease course. Third, considering that the visual field test might be inaccurate during the inflammation attack of PSS, we did not perform visual field test, and thus no such data were presented in this study.

In conclusion, we found significant iris atrophy in PSS-affected eyes. Moreover, iris stromal thickness close to the pupillary edge was significantly associated with corneal endothelium density, C/D ratio, and RNFL thickness in PSS-affected eyes, indicating that iris stromal thickness close to the pupillary edge could be a novel clinical predictor of the changes in corneal endothelium, C/D ratio, and RNFL thickness in PSS.

## Figures and Tables

**Table 1:** Study subject characteristics.

	Affected Eye	Fellow Eye	*p*
Age (years)	36.56 ± 11.80	36.56 ± 11.80	-
Sex (Male/Female)	34/27	34/27	-
Axial length (mm)	23.88 ± 0.95	23.87 ± 0.91	0.848
Corneal endothelium density (/mm^2^)	2447.70 ± 423.93	2706.43 ± 297.06	<0.001 *
C/D ratio	0.50 ± 0.19	0.37 ± 0.09	<0.001 *
Retinal nerve fiber layer thickness (μm)	94.56 ± 23.34	101.72 ± 10.38	0.002 *
Intraocular pressure (mmHg)	23.71 ± 11.96	17.43 ± 3.74	<0.001 *
Pupil diameter (mm)	4.63 ± 0.88	4.43 ± 0.89	0.032 *

* Paired *t*-test.

**Table 2:** Univariate linear regression of associations of iris thickness and corneal endothelium density in PSS-affected eyes.

Affected Eye	Corneal Endothelium Density (/mm^2^)							
	*β*	*p*		*β*	*p*		*β*	*p*
A500 (μm)	0.441	0.740	M500 (μm)	0.538	0.047 *	P500 (μm)	−0.656	0.850
A1000 (μm)	−0.161	0.938	M1000 (μm)	0.827	0.018 *	P1000 (μm)	3.737	0.209
A1500 (μm)	2.140	0.382	M1500 (μm)	0.227	0.713	P1500 (μm)	3.262	0.268
A2000 (μm)	2.913	0.237	M2000 (μm)	0.317	0.639	P2000 (μm)	2.324	0.480
A2500 (μm)	1.216	0.412	M2500 (μm)	0.805	0.175	P2500 (μm)	4.922	0.096

*β/p* values: regression coefficient and *p* values of the independent variables in generalized estimating equations. The influence factors as age, sex, AL, PD, and IOP have been adjusted. * Generalized estimating equations. A: The anterior border layer, M: the middle stromal layer, P: the posterior pigmented epithelial layer.

**Table 3:** Univariate linear regression of associations of iris thickness and C/D ratio in PSS-affected eyes.

Affected Eye	C/D Ratio							
	*β*	*p*		*β*	*p*		*β*	*p*
A500 (μm)	0.0001	0.859	M500 (μm)	−0.0005	0.001 *	P500 (μm)	0.0004	0.818
A1000 (μm)	0.0005	0.548	M1000 (μm)	−0.0002	0.407	P1000 (μm)	−0.0005	0.770
A1500 (μm)	0.0007	0.546	M1500 (μm)	0.0006	0.200	P1500 (μm)	0.0001	0.968
A2000 (μm)	−0.0002	0.851	M2000 (μm)	0.0004	0.138	P2000 (μm)	0.0001	0.955
A2500 (μm)	−0.0013	0.055	M2500 (μm)	0.0004	0.143	P2500 (μm)	−0.0014	0.246

*β/p* values: regression coefficient and *p* values of the independent variables in generalized estimating equations. The influence factors as age, sex, AL, PD, and IOP have been adjusted. * Generalized estimating equations. A: The anterior border layer, M: the middle stromal layer, P: the posterior pigmented epithelial layer.

**Table 4:** Univariate linear regression of associations of iris thickness and RNFL thickness in PSS-affected eyes.

Affected Eye	RNFL Thickness (μm)							
	*β*	*p*		*β*	*p*		*β*	*p*
A500 (μm)	−0.060	0.415	M500 (μm)	0.043	0.037 *	P500 (μm)	−0.112	0.541
A1000 (μm)	−0.092	0.288	M1000 (μm)	0.005	0.824	P1000 (μm)	0.027	0.890
A1500 (μm)	−0.022	0.873	M1500 (μm)	−0.071	0.100	P1500 (μm)	0.009	0.956
A2000 (μm)	−0.046	0.734	M2000 (μm)	−0.023	0.466	P2000 (μm)	−0.066	0.674
A2500 (μm)	0.201	0.058	M2500 (μm)	−0.027	0.415	P2500 (μm)	0.091	0.601

*β/p* values: regression coefficient and *p* values of the independent variables in generalized estimating equations. The influence factors as age, sex, AL, PD, and IOP have been adjusted. * Generalized estimating equations. A: The anterior border layer, M: the middle stromal layer, P: the posterior pigmented epithelial layer.

**Table 5:** Univariate linear regression of associations of iris thickness and IOP in PSS-affected and fellow eyes.

**Affected Eye**	**IOP (mmHg)**							
	***β***	***p***		***β***	***p***		***β***	***p***
A500 (μm)	−0.028	0.514	M500 (μm)	0.004	0.783	P500 (μm)	−0.053	0.591
A1000 (μm)	−0.059	0.208	M1000 (μm)	−0.004	0.803	P1000 (μm)	−0.114	0.356
A1500 (μm)	0.135	0.065	M1500 (μm)	0.006	0.742	P1500 (μm)	−0.016	0.848
A2000 (μm)	0.035	0.598	M2000 (μm)	0.004	0.844	P2000 (μm)	0.036	0.787
A2500 (μm)	−0.090	0.209	M2500 (μm)	−0.013	0.513	P2500 (μm)	0.042	0.660
**Fellow Eye**	**IOP (mmHg)**							
	***β***	***p***		***β***	***p***		***β***	***p***
A500 (μm)	−0.009	0.406	M500 (μm)	−0.003	0.519	P500 (μm)	0.023	0.309
A1000 (μm)	−0.041	0.200	M1000 (μm)	−0.008	0.087	P1000 (μm)	−0.036	0.120
A1500 (μm)	−0.029	0.213	M1500 (μm)	−0.008	0.078	P1500 (μm)	−0.017	0.640
A2000 (μm)	−0.021	0.132	M2000 (μm)	−0.008	0.098	P2000 (μm)	−0.022	0.429
A2500 (μm)	−0.012	0.550	M2500 (μm)	−0.008	0.199	P2500 (μm)	−0.022	0.386

*β*/*p* values: regression coefficient and *p* values of the independent variables in generalized estimating equations. The influence factors as age, sex, AL, and PD have been adjusted. A: The anterior border layer, M: the middle stromal layer, P: the posterior pigmented epithelial layer.

## Data Availability

The data that support the findings of this study are available from the corresponding author upon reasonable request.
